# Robotic assisted vs. open ureteral reimplantation in adults: a systematic review and meta-analysis

**DOI:** 10.1007/s11701-025-02511-1

**Published:** 2025-07-11

**Authors:** Atef A. Hassan, Abdelrahman M. Mady, Hesham Abozied, Mohamed I. Algammal, Amr A. Hassan, Mohamed Salman, Mohamed E. Metwally, Moaz Abouelmagd, Hossam A. Shouman, Ibrahim Tagreda, Mohamed Elsalhy, Esam Elnady, Mohamed Rehan, Saed Khater

**Affiliations:** 1 Faculty of Medicine, Al-Azhar University, Cairo, Egypt; 2 Department of Urology, Faculty of Medicine, Al-Azhar University, Cairo, Egypt; 3 Faculty of Medicine, Cairo University, Cairo, Egypt; 4 Department of Urology, Faculty of Medicine, Al-Azhar University, Damietta, Egypt

**Keywords:** Open ureteral reimplantation, Robotic-assisted ureteral reimplantation, Ureteral strictures, Ureteral surgery

## Abstract

Ureteral reimplantation is a critical surgical procedure for restoring ureteral function, traditionally performed using open ureteral reimplantation (OUR). However, robotic-assisted ureteral reimplantation (RUR) has emerged as a minimally invasive alternative with potential perioperative advantages. To systematically compare the postoperative outcomes and efficacy of RUR and OUR in ureteral reimplantation in adults through a meta-analysis of existing studies. A comprehensive search of PubMed, Scopus, Web of Science, and the Cochrane Library identified studies comparing RUR and OUR in adults up to January 2025. Studies reporting at least one outcome of interest, such as complications, blood loss, hospital stay, or reintervention rates, were included. Pooled effect estimates were calculated using a random-effects model, and heterogeneity was assessed using the I^2^ statistic. Four retrospective studies encompassing 258 patients (RUR: 141, OUR: 117) were included. RUR was associated with significantly fewer complications (RR: 0.40; 95% CI: 0.17–0.91; *p* = 0.03), shorter hospital stays (MD: −4.97 days; 95% CI: −9.55 to −0.38; *p* = 0.03), and reduced transfusion requirements (RR: 0.09; 95% CI: 0.02–0.46; *p* = 0.004) compared to OUR. No significant differences were observed in reintervention rates or operating time. RUR demonstrates significant perioperative advantages over OUR, including reduced complications, shorter hospital stays, and lower blood loss, while maintaining comparable long-term efficacy. These findings support the adoption of RUR as a safe and effective alternative for ureteral reimplantation. Further high-quality, multicenter studies are needed to confirm these results and address remaining gaps.

**Supplementary Information:**

The online version contains supplementary material available at 10.1007/s11701-025-02511-1.

## Introduction

Ureteral reimplantation is a vital surgical procedure aimed at restoring ureteral function following strictures or injuries, conditions often resulting from malignancy, prior surgeries, or other anatomical challenges [1]. Traditionally, open ureteral reimplantation (OUR) has been the gold standard for managing these cases, offering proven durability and long-term success in preventing the recurrence of obstruction or stricture. However, the procedure is associated with significant morbidity, including longer hospital stays, higher postoperative pain, and extended recovery times [2]. With the rapid advancements in minimally invasive surgery, robotic-assisted ureteral reimplantation (RUR) has emerged as a compelling alternative [3]. The clinical management of uretero-enteric strictures and other ureteral pathologies is further complicated by patient-specific factors, such as comorbid conditions, anatomical variations, and the presence of prior radiation or extensive adhesions [4].

Robotic-assisted surgery offers several technical advantages over the open approach, including enhanced precision due to three-dimensional visualization, improved dexterity, and reduced surgeon fatigue through ergonomic operating platforms [5, 6]. These advancements have translated into clinical benefits, with studies reporting lower blood loss, shorter hospital stays, and fewer perioperative complications compared to traditional open surgery. Despite these advantages, concerns remain regarding the learning curve, cost-effectiveness, and long-term durability of robotic-assisted approaches [7, 8].

In addition to the technical and clinical benefits of robotic-assisted surgery, there is growing interest in its cost-effectiveness and impact on healthcare systems. While initial investment and operational costs of robotic systems are significant, proponents argue that these costs may be offset by reduced hospital stays, fewer complications, and quicker recovery times, ultimately improving patient satisfaction and reducing long-term healthcare expenditures [9]. Moreover, the evolving technology of robotic platforms, including enhanced surgical training modules and artificial intelligence integration, holds promise for further optimizing outcomes in ureteral reimplantation and other surgical domains [6]. These advancements underscore the importance of systematically evaluating the comparative efficacy of robotic-assisted and open approaches to inform evidence-based clinical decision-making and policy development [10].

While both robotic and open approaches have shown efficacy, comparative evidence remains fragmented, with some studies highlighting the superior perioperative outcomes of robotic surgery and others underscoring the durability and reliability of open surgery [7, 11, 12].

This meta-analysis seeks to address these gaps by systematically synthesizing evidence from published studies comparing robotic-assisted and OUR in adults. This analysis aims to provide a comprehensive understanding of the relative benefits and limitations of each approach.

## Methods

The present investigation adhered to the methodologies described in the Cochrane Handbook of Systematic Reviews on Interventions [13]. The publication was meticulously prepared following the requirements of the Preferred Reporting Items for Systematic Reviews and Meta-Analyses (PRISMA) statement [14]. Additionally, the work was reported in accordance with the guidelines of AMSTAR-2 (Assessing the Methodological Quality of Systematic Reviews [15]). The protocol was retrospectively registered in PROSPERO in accordance with PRISMA guidelines to ensure transparency and methodological rigor.

### Literature searching and study selection

The screening and selection process for this meta-analysis followed a structured and systematic approach, adhering to the guidelines set forth by the Preferred Reporting Items for Systematic Reviews and Meta-Analyses (PRISMA) [14]. We conducted a comprehensive search across four major electronic databases: PubMed, Scopus, Web of Science, and the Cochrane Library, covering all studies published up until January 10, 2025. The search strategy included terms related to RUR and OUR. The complete search strategy for each database is provided in Supplementary Table 1. All retrieved records were imported into EndNote for organization and deduplication. Two independent reviewers conducted a two-step screening process. Initially, titles and abstracts were screened to exclude irrelevant studies. The remaining full-text articles were assessed for eligibility based on predefined inclusion and exclusion criteria. Reference lists of included studies and relevant reviews were also screened manually to identify additional eligible studies. Furthermore, to minimize selection bias, a dual-review process was employed at each stage of study selection and data extraction, with discrepancies resolved through consensus or third-party adjudication.

### Requirements for eligibility

The inclusion criteria for this meta-analysis required studies to directly compare RUR and OUR while reporting at least one outcome of interest, such as complications, operative time, blood loss, hospital stay, reintervention rates, or stricture recurrence. Eligible studies included retrospective and prospective cohort studies as well as randomized controlled trials (RCTs). Studies were excluded if they lacked sufficient data for outcome extraction or analysis, were limited to conference abstracts, case reports, case series, or non-human research, or were published in non-English languages.

### Quality assessment

The quality of the retrospective studies was assessed according to the Newcastle–Ottawa quality assessment scale [16]. Two authors independently evaluated the quality of the included studies, and in case of any disagreement, the first author took the final decision.

### Data extraction

Data extraction was performed independently by two reviewers using a standardized data extraction form. For each included study, the following information was collected: study characteristics (author, year, country, study design, and setting), participant demographics [sample size, mean or median age, gender distribution, and body mass index (BMI)], clinical characteristics (ASA score, comorbidities, and follow-up duration), and surgical details (operative time, blood loss, complications, hospital stay, and reintervention rates).

### Statistical analysis

Statistical analyses were conducted using Review Manager (RevMan, version 5.4, The Cochrane Collaboration). For continuous outcomes, mean differences (MD) with 95% confidence intervals (CI) were calculated. For dichotomous outcomes, relative risks (RR) with 95% CI were reported. Heterogeneity across studies was assessed using the Chi-squared (χ^2^) test and quantified with the I^2^ statistic. Heterogeneity was categorized as low (I^2^ = 0%–25%), moderate (I^2^ = 26%–50%), or high (I^2^ > 50%). A random-effects model was applied. To assess the robustness of the results, sensitivity analyses were performed by excluding individual studies one at a time to determine their influence on pooled estimates.

## Results

### Literature search

A comprehensive literature search was conducted across four databases: PubMed, Scopus, Web of Science (WOS), and the Cochrane Library, to identify studies comparing RUR to OUR. The search yielded 64 results from PubMed, 116 from Scopus, 76 from WOS, and one from the Cochrane Library, for a total of 257 studies.

**Fig. 1:**
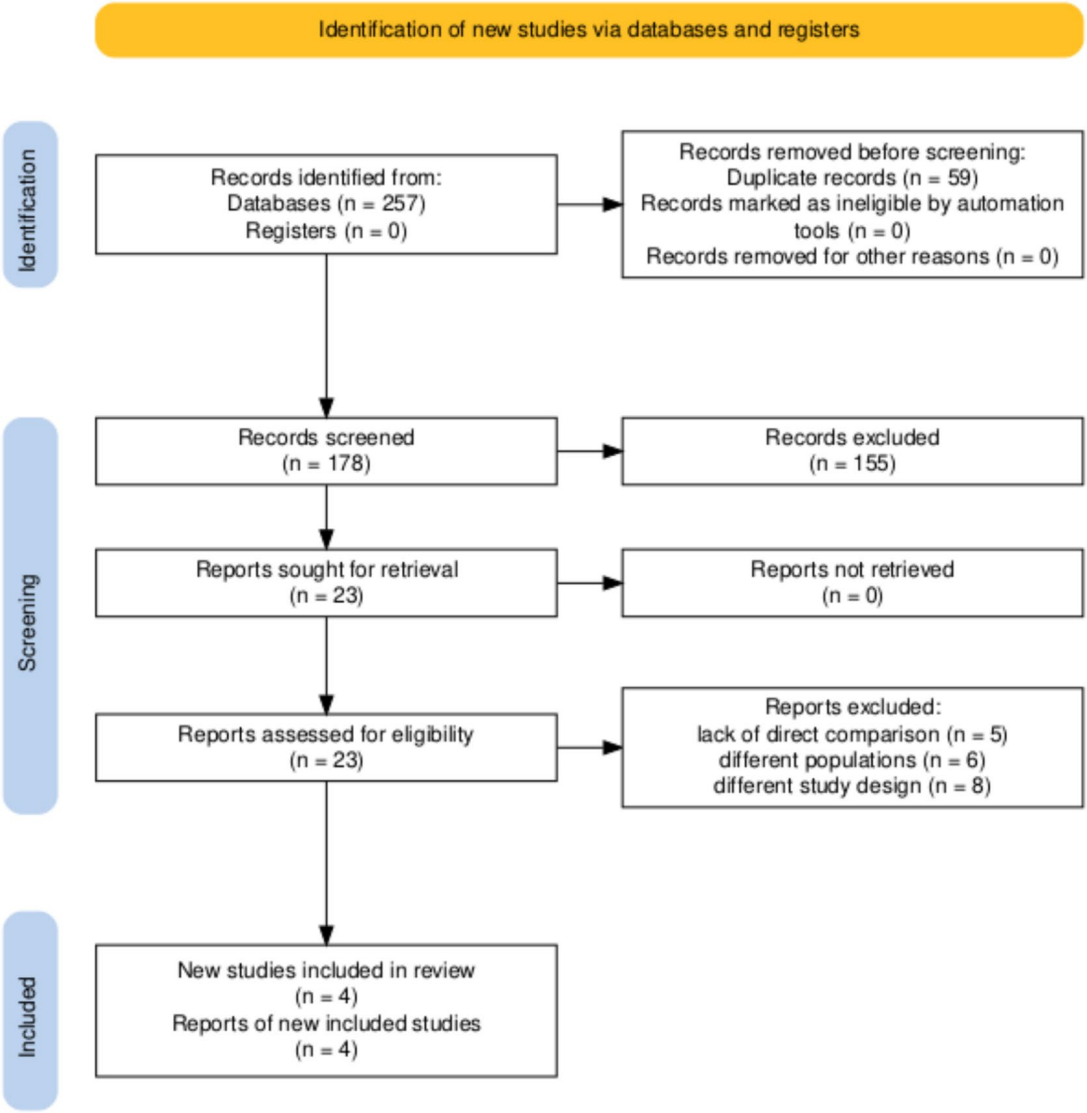
PRISMA flow diagram of the included studies

After removing duplicates, 198 unique studies remained. Titles and abstracts of these studies were screened for relevance, resulting in the exclusion of 175 studies based on predefined eligibility criteria. Twenty-three studies underwent full-text review, of which 19 were excluded for reasons such as lack of direct comparison between RUR and OUR or different populations. Ultimately, four studies met all inclusion criteria and were included in the meta-analysis. The selection process is visualized in the PRISMA flow diagram Fig. 1

### Characteristics of the included studies

Table 1 summarizes the characteristics of the included studies. Four studies were analyzed, encompassing a total of 258 patients, with 141 undergoing RUR and 117 undergoing OUR. The included studies were conducted in the USA and internationally, with two single-centre studies from the USA and two multicenter studies providing broader, international data. All included studies were retrospective in design. The median or mean age of participants ranged from 55 to 67 years, with the proportion of male patients varying between 51% and 85.7%. Mean body mass index (BMI) was reported in all studies, ranging from 24.9 to 28.5. Comorbidity profiles, as measured by the American Society of Anesthesiologists (ASA) score, indicated that the majority of patients fell within ASA I–III, with higher proportions of ASA III in the RUR group in some studies. Follow-up durations ranged from 12 to 23.5 months.

**Table 1:** Characteristics of the included studies

Study (References)	Country	Sample size (RUR/OUR)	Study design	Population	Age (mean/median)	Sex (% male)	BMI (mean)	Comorbidities (ASA score)	Follow-up (months)	Indications for reimplantation	Surgical techniques
[11]	USA	18/16	Retrospective	Post-radical cystectomy	67 (median)	75	28.5	ASA II–III	23 (median)	Cystectomy, attributed to poor vascularity and suboptimal technique	Robotic-assisted ureteroenteric anastomosis performed with tension-free technique using a running Vicryl suture. Anastomosis site was reinforced with omental wrapping. Ureteral stents were routinely placed for 6 weeks
[7]	Multicenter	65/17	Retrospective	Benign UES	66.2 (mean)	83	28.3	ASA III (47.7% RUR)	23.5 (median)	Benign ureteroenteric strictures (UES) post-radical cystectomy, with imaging-confirmed diagnoses (CT, pyelogram)	Resection of the strictured ureteral segment followed by a tension-free robotic-assisted ureteroenteric reimplantation. Anastomoses were created with a running 3–0 Vicryl suture and routinely checked intraoperatively for leaks with methylene blue dye
[17]​	USA	5/7	Retrospective	Post-cystectomy UAS	59.5 (median)	85.70	27.8	ASA not reported	12 (median)	Ureteroenteric strictures after cystectomy; included patients with failed prior endoscopic treatments	Robotic-assisted ureteroenteric reimplantation with careful dissection of fibrotic tissue around the ureter. The anastomosis was created with interrupted sutures for proximal patency. Omental pedicle flaps were used for reinforcement in high-risk cases
[8]	Multicenter	51/79	Retrospective	Ureteral injury	55 (median)	51	24.9	ASA I–II (majority)	14 (median)	Ureteral injuries due to prior surgeries, radiation-induced strictures, and tumor-related obstructions	Reconstruction of ureteral injuries using robotic-assisted ureteroureterostomy or ureteroneocystostomy depending on the location of injury. Ureteral spatulation was performed to optimize the anastomotic surface. Ureteral stents were used in all cases

### Quality of the included studies

**Fig. 2:**
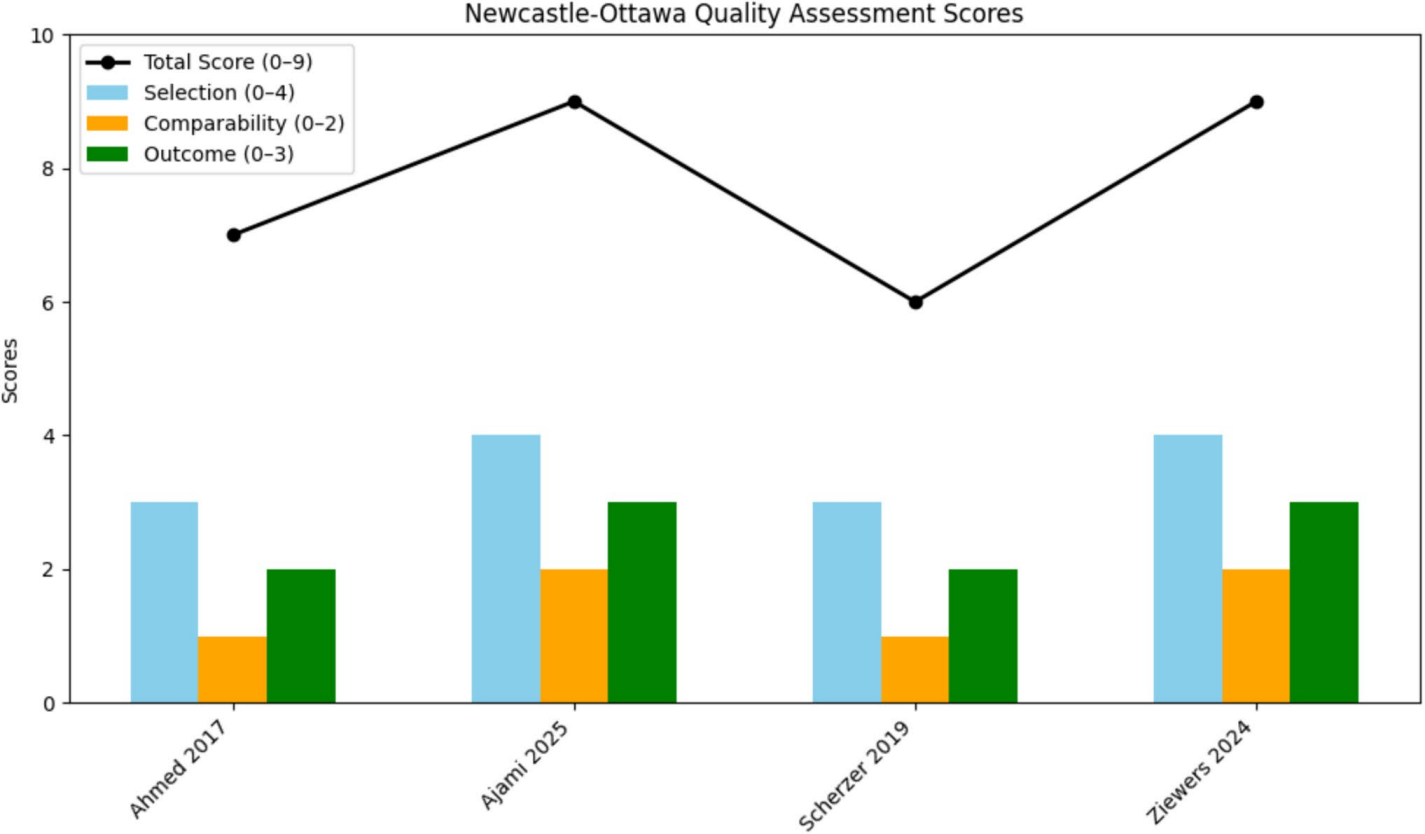
Methodological quality across the studies, according to the Newcastle–Ottawa scale

The total scores ranged from 6 to 9, indicating moderate to high methodological quality across the studies, according to the Newcastle–Ottawa Scale (Fig. 2)*.* Two studies ([7] and [8]) achieved the maximum score of 9, reflecting robust designs, comprehensive adjustment for confounders, and clear outcome definitions. These studies also featured multicenter data, enhancing generalizability. One study ([11]) scored 7, with a strong selection process but limited adjustment for confounders and minor loss to follow-up. Scherzer et al. [17] had a score of 6, primarily due to a lack of comprehensive adjustment for confounders and some loss to follow. In the selection domain, all studies demonstrated representativeness of the cohort and reliable exposure ascertainment, with multicenter designs contributing additional points in two studies. For comparability, only two studies fully adjusted for key confounders, such as ASA scores and prior surgeries. In the domain, follow-up adequacy and clearly defined outcomes were present in all studies, although some studies showed minor losses to follow-up.

### Clinical outcomes

#### Need for reintervention

**Fig. 3:**
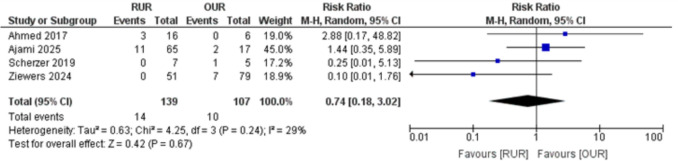
Forest plot of need for reintervention

The pooled analysis of four studies evaluating the need for reintervention demonstrated no statistically significant difference between RUR and OUR. The RR was 1.25 (95% CI: 0.39 to 4.00; *p* = 0.71), with low heterogeneity (I^2^ = 29%), Fig. 3*.*

**Fig. 4:**
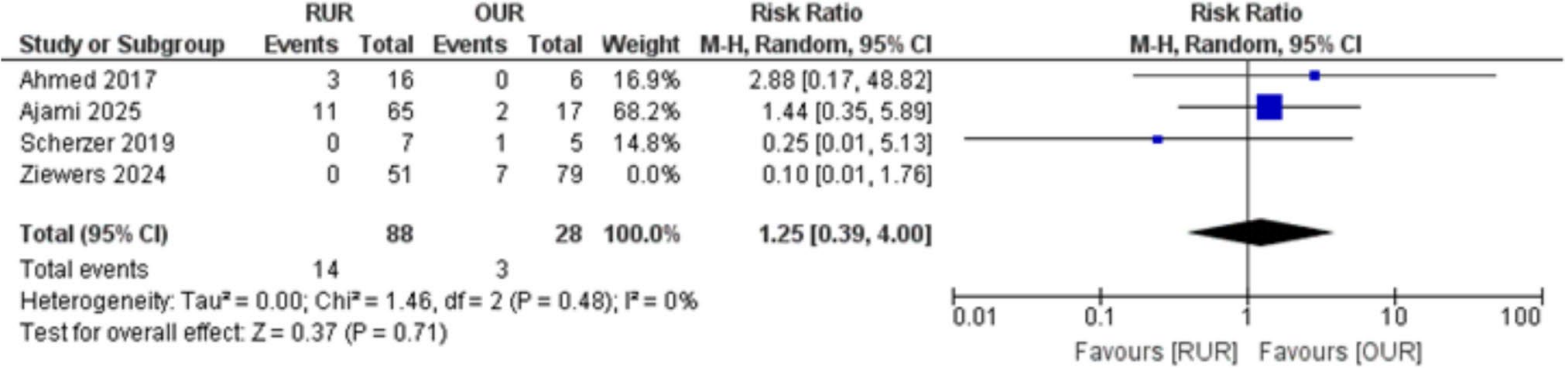
Forest plot of need for reintervention after excluding Ziewers 2024

A sensitivity analysis excluding Ziewers et al. [8] resolved the heterogeneity (I^2^ = 0%). The recalculated RR was 0.74 (95% CI: 0.18 to 3.02; *p* = 0.67), indicating no statistically significant difference between RUR and OUR in the need for reintervention, Fig. 4.

#### Complications

**Fig. 5:**
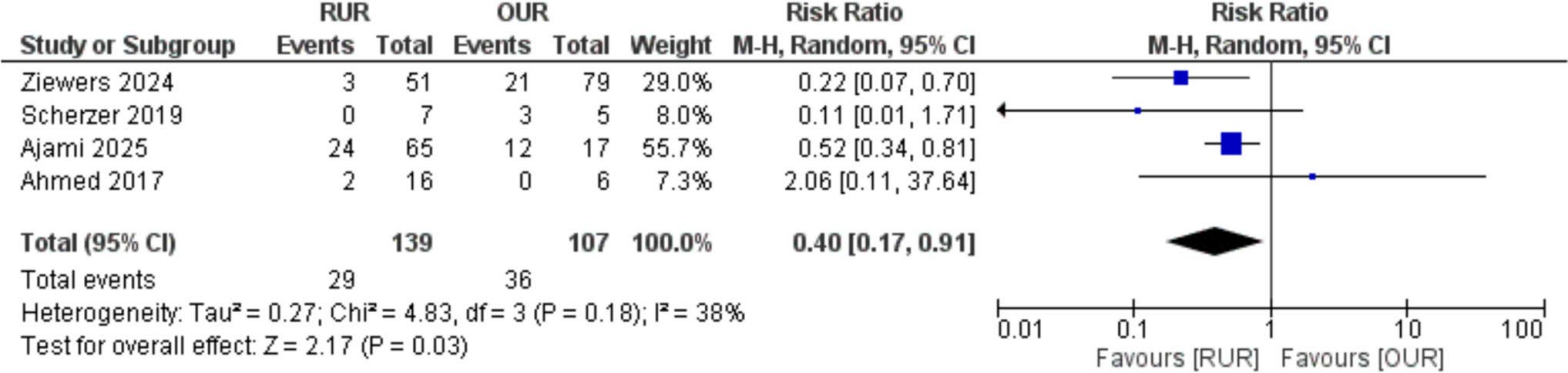
Forest plot of complications

Four studies assessed complications. RUR was associated with a significantly lower risk of complications compared to OUR (RR = 0.40; 95% CI: 0.17–0.91; *p* = 0.03). Heterogeneity was moderate (I^2^ = 38%), indicating some variability across studies, Fig. 5.

#### Hospital stay

**Fig. 6:**
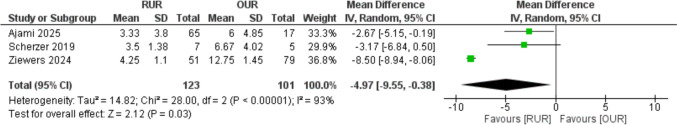
Forest plot of hospital stay

Patients undergoing RUR had a significantly shorter hospital stay compared to OUR (MD = −4.97 days; 95% CI: −9.55 to −0.38; *p* = 0.03). Substantial heterogeneity was observed (I^2^ = 93%), likely due to differences in study populations and institutional practices, Fig. 6.

#### Operative time

**Fig. 7:**
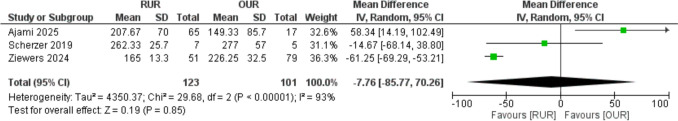
Forest plot of operative time

There was no significant difference in operative time between RUR and OUR (MD = −7.76 min; 95% CI: −85.77 to 70.26; *p* = 0.85). Substantial heterogeneity (I^2^ = 93%) reflected variations in surgical complexity and techniques, Fig. 7.

#### Need for blood transfusion

**Fig. 8:**
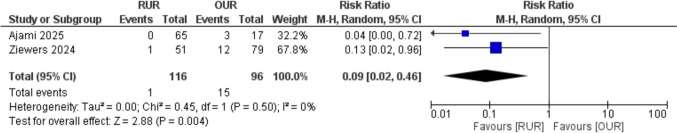
Forest plot of need for blood transfusion

Two studies reported on transfusion requirements. RUR significantly reduced the need for transfusion compared to OUR (RR = 0.09; 95% CI: 0.02–0.46; *p* = 0.004), with no observed heterogeneity (I^2^ = 0%), Fig. 8.

#### Estimated blood loss

**Fig. 9:**
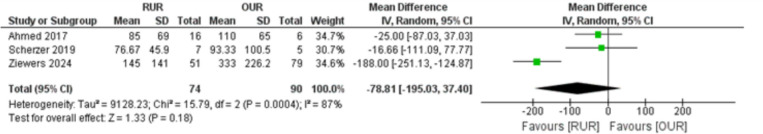
Forest plot of estimated blood loss

The pooled analysis of estimated blood loss included three studies, with a mean difference (MD) of −78.81 mL (95% CI: −195.03 to 37.40; *p* = 0.18). High heterogeneity was observed (I^2^ = 87%), indicating variability among the included studies, Fig. 9.

**Fig. 10:**
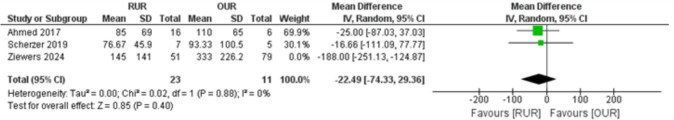
Forest plot of estimated blood loss after excluding Ziewers 2024

To resolve this heterogeneity, a leave-one-out sensitivity analysis was performed. Excluding Ziewers et al. [8] reduced heterogeneity to I^2^ = 0%, with a recalculated MD of −22.49 mL (95% CI: −74.33 to 29.36; *p* = 0.40). The results remained statistically non-significant, suggesting no meaningful difference in EBL between RUR and OUR, Fig. 10.

## Discussion

This meta-analysis is the first to comprehensively compare RUR and OUR for ureteral reimplantation. By synthesizing data from four studies, we evaluated key outcomes including postoperative complications, estimated blood loss, length of hospital stay, and reintervention rates. This meta-analysis demonstrated that RUR was associated with a significantly lower incidence of postoperative complications, reduced blood loss, and shorter hospital stays compared to OUR. Additionally, no significant difference was observed in reintervention rates between the two approaches, indicating comparable long-term durability. These findings highlight the overall perioperative advantages of RUR while maintaining similar efficacy to OUR in ureteral reimplantation.

Our findings indicated no significant difference in the need for reintervention between RUR and OUR, suggesting comparable long-term durability of the two approaches. This result aligns with Ahmed et al. [11], who reported similar reintervention rates between robotic and open revisions for ureteroenteric strictures​. However, Scherzer et al. [17] emphasized that robotic techniques may be better suited for early-stage strictures due to their minimally invasive nature​. The meta-analysis by Yang et al. [18] provides a comprehensive evaluation of robotic ureteral reconstruction for benign ureteral strictures, highlighting its advantages over open and laparoscopic approaches. Their findings indicate that robotic reconstruction is associated with significantly lower estimated blood loss and shorter hospital stays compared to open surgery, as well as reduced operative time when compared to laparoscopic procedures. However, we are focusing on reimplantation only, these results align with our observations, reinforcing the perioperative benefits of RUR. However, Yang et al. [18] also emphasizes the need for further studies to determine the superiority of RUR conclusively. This underscores the importance of our current analysis, which aims to contribute additional evidence to this evolving field. Variability in patient selection and surgical expertise likely contributes to the observed heterogeneity in this outcome.

Our analysis demonstrated that RUR was associated with a significantly lower incidence of postoperative complications compared to OUR. This finding aligns with Ziewers et al. [8], who reported that RUR had a complication rate of 5.9% compared to 26.6% in the OUR group, attributing the reduced complications to the minimally invasive nature of robotic surgery​. Similarly, Ajami et al. [7] observed significantly fewer high-grade complications (Clavien III–IV) with RUR compared to OUR​. The enhanced precision of robotic platforms, improved visualization, and reduced trauma to surrounding tissues may explain the superior outcomes of RUR. However, variability in the reporting of complications across studies could account for residual heterogeneity in this analysis, underscoring the need for standardized reporting in future research.

While our pooled analysis initially showed high heterogeneity, sensitivity testing revealed a trend towards reduced blood loss with RUR. Ziewers et al. [8] found significantly lower blood loss in RUR (median 150 mL) compared to OUR (median 300 mL), a difference attributed to the improved hemostatic control offered by robotic platforms​. Similar trends were noted by Ajami et al. [7], emphasizing the benefits of robotic surgery in reducing intraoperative trauma​. These findings are clinically relevant, as reduced blood loss translates into fewer transfusion requirements and faster recovery.

Patients undergoing RUR experienced a significantly shorter hospital stay compared to those undergoing OUR, a finding consistent with previous reports. Ziewers et al. [8] reported a median hospital stay of 4 days for RUR versus 14 days for OUR, while Ajami et al. [7] observed similarly shorter hospitalization for robotic surgery​​. This outcome reflects the minimally invasive nature of RUR, which reduces postoperative pain, accelerates recovery, and minimizes the risk of nosocomial infections. The variation in hospital stay across studies could be influenced by institutional practices and discharge criteria, emphasizing the importance of contextualizing these results within local healthcare settings.

### Strengths

This meta-analysis synthesizes evidence from multiple studies, providing a comprehensive comparison of RUR and OUR. The inclusion of studies with diverse patient populations and surgical settings enhances the generalizability of our findings. Additionally, our rigorous methodology, including sensitivity analyses and heterogeneity assessment, ensures the robustness of the results. These strengths underscore the value of this analysis in addressing critical gaps in literature and guiding clinical practice.

### Limitations

Several limitations should be acknowledged. First, the included studies were all retrospective, introducing potential biases in data collection and reporting. This design inherently carries a risk of selection bias and limits the ability to control for confounding variables. Although the included studies received moderate to high NOS scores (6–9), unadjusted factors such as baseline differences in patient comorbidities, surgeon experience, and institutional protocols may have influenced the reported outcomes. The absence of propensity score matching or multivariate adjustment in some studies further limits the strength of the conclusions. These methodological limitations must be considered when interpreting the perioperative advantages observed with RUR.

Second, the small sample sizes and variability in study designs limit the statistical power of our analysis. While our meta-analysis demonstrated a significantly lower transfusion requirement in the RUR group (RR = 0.09; 95% CI: 0.02–0.46), this result was derived from only two studies with relatively small sample sizes. Therefore, caution is warranted when interpreting this finding, as it may not be generalizable. Moreover, although the mean estimated blood loss was numerically lower in the RUR group (MD = −78.81 mL), this difference did not reach statistical significance (*p* = 0.18). These findings suggest a potential but not definitive advantage in blood conservation with RUR. Accordingly, while RUR may offer perioperative benefits, especially in selected cases, these conclusions should be considered preliminary and hypothesis-generating rather than definitive.

Third, differences in surgeon experience and institutional practices likely contributed to heterogeneity in some outcomes. Specifically, the high heterogeneity observed in operative time (I^2^ = 93%) and length of hospital stay (I^2^ = 93%) may reflect variability in surgical techniques (e.g., extent of dissection, use of intraoperative stents), perioperative management protocols (e.g., ERAS programs, catheter management), patient selection criteria, and hospital discharge policies across the included centers. Additionally, differences in the learning curve associated with robotic surgery may have influenced operative efficiency. While sensitivity analyses confirmed the stability of pooled estimates, these sources of heterogeneity must be considered when interpreting the results.

Fourth, the absence of subgroup analyses based on the indications for reimplantation may contribute to heterogeneity in the results. The studies included did not provide sufficient data to support such analyses. To overcome these limitations, future research should prioritize multicenter randomized controlled trials with standardized outcome reporting.

Additionally, cost-effectiveness analyses and evaluations of patient-reported outcomes are essential to provide a more holistic comparison of RUR and OUR. The high cost of robotic platforms, including acquisition, maintenance, and operative consumables, remains a significant barrier to widespread adoption. Although our analysis suggests shorter hospital stays and fewer complications with RUR, the lack of formal economic evaluation limits our ability to determine whether these clinical advantages translate into overall cost savings. Future studies should incorporate comprehensive cost-effectiveness models to guide evidence-based resource allocation in surgical practice.

## Conclusion

In conclusion, our meta-analysis demonstrates that RUR offers significant advantages over OUR, including reduced complications, shorter hospital stays, and lower blood loss, while maintaining comparable long-term efficacy. These findings highlight the potential of RUR as a safe and effective alternative to OUR for ureteral reimplantation. However, given the retrospective nature of the included studies and the limited median follow-up durations (12–23.5 months), the long-term efficacy and durability of RUR remain to be fully established. Consequently, further high-quality research is needed to validate these results and address remaining knowledge gaps.

## Supplementary Information

Below is the link to the electronic supplementary material.

Supplementary file1 (DOCX 15 KB)

## Data Availability

No datasets were generated or analysed during the current study.
