# Supplementary material for: Robotic assisted vs. open ureteral reimplantation in adults: a systematic review and meta-analysis
Source: J Robot Surg. 2025 Jul 11;19(1):373. doi: 10.1007/s11701-025-02511-1 (PMC12254083; doi:10.1007/s11701-025-02511-1)
Supplement: Supplementary file 1 — Supplementary file1 (DOCX 15 KB) [file 11701_2025_2511_MOESM1_ESM.docx]

| Supplementary Table 1. Search strategy for each database | |
| --- | --- |
| Database | **Search strategy** |
| PubMed | ("robotic-assisted" OR "robotic surgery" OR "robot-assisted" OR "robotic") AND ("ureteral reimplantation" OR "ureteral reconstruction" OR "ureteral surgery") AND ("open surgery" OR "open reimplantation" OR "open ureteral repair") |
| Scopus | ("robotic-assisted" OR "robotic surgery" OR "robot-assisted" OR "robotic") AND ("ureteral reimplantation" OR "ureteral reconstruction" OR "ureteral surgery") AND ("open surgery" OR "open reimplantation" OR "open ureteral repair") |
| Web of Science | ("robotic-assisted" OR "robotic surgery" OR "robot-assisted" OR "robotic") AND ("ureteral reimplantation" OR "ureteral reconstruction" OR "ureteral surgery") AND ("open surgery" OR "open reimplantation" OR "open ureteral repair") |
| Cochrane Liberary | (robotic-assisted OR robotic surgery OR robot-assisted OR robotic) AND (ureteral reimplantation OR ureteral reconstruction OR ureteral surgery) AND (open surgery OR open reimplantation OR open ureteral repair) |
